# Pedigree based DNA sequencing pipeline for germline genomes of cancer families

**DOI:** 10.1186/s13053-016-0058-1

**Published:** 2016-08-09

**Authors:** Asta Försti, Abhishek Kumar, Nagarajan Paramasivam, Matthias Schlesner, Calogerina Catalano, Dagmara Dymerska, Jan Lubinski, Roland Eils, Kari Hemminki

**Affiliations:** 1 Division of Molecular Genetic Epidemiology, German Cancer Research Center (DKFZ), D69120 Heidelberg, Germany; 2 Center for Primary Health Care Research, Lund University, Malmö, Sweden; 3 Division of Theoretical Bioinformatics, German Cancer Research Center (DKFZ), D69120 Heidelberg, Germany; 4 Medical Faculty Heidelberg, Heidelberg University, Heidelberg, Germany; 5 Hereditary Cancer Center, Pomeranian Medical University, Szczecin, Poland; 6 Department of Bioinformatics and Functional Genomics, Institute of Pharmacy and Molecular Biotechnology (IPMB) and BioQuant, Heidelberg University, Heidelberg, Germany

**Keywords:** Mutation, Family-based, Germline genetics, Genetic risk factors

## Abstract

**Background:**

In the course of our whole-genome sequencing efforts, we have developed a pipeline for analyzing germline genomes from Mendelian types of cancer pedigrees (familial cancer variant prioritization pipeline, FCVPP).

**Results:**

The variant calling step distinguishes two types of genomic variants: single nucleotide variants (SNVs) and indels, which undergo technical quality control. Mendelian types of variants are assumed to be rare and variants with frequencies higher that 0.1 % are screened out using human 1000 Genomes (Phase 3) and non-TCGA ExAC population data. Segregation in the pedigree allows variants to be present in affected family members and not in old, unaffected ones. The effectiveness of variant segregation depends on the number and relatedness of the family members: if over 5 third-degree (or more distant) relatives are available, the experience has shown that the number of likely variants is reduced from many hundreds to a few tens. These are then subjected to bioinformatics analysis, starting with the combined annotation dependent depletion (CADD) tool, which predicts the likelihood of the variant being deleterious. Different sets of individual tools are used for further evaluation of the deleteriousness of coding variants, 5’ and 3’ untranslated regions (UTRs), and intergenic variants.

**Conlusions:**

The likelihood of success of the present genomic pipeline in finding novel high- or medium-penetrant genes depends on many steps but first and foremost, the pedigree needs to be reasonably large and the assignments and diagnoses among the members need to be correct.

## Background

The application of next-generation sequencing (NGS) has hugely increased the number of detected somatic mutations in human cancers. Even though NGS would afford an advantageous technology also for germline sequencing, no boost in the number of new cancer predisposing genes has been evident. When Dr. Rahman surveyed the discovery of 114 cancer predisposing genes until year 2014, only 6 genes were reported to be found by ‘genome-wide mutation analysis’ [1]. That review also showed that the most successful period of finding predisposing genes was the latter part of the 1990s when family/pedigree-based linkage analysis was the main genetic approach. In fact, the few recent successes in gene finding in the germline, such as NTHL1 in colorectal cancer or RECQL in breast cancer, were not pedigree-based even though focusing on cancer families [2, 3]. Family-based studies a priori are statistically more powerful than those based on sporadic cases and they may find known high-risk mutations but for novel rare predisposing genes external validation would be needed as shown in previous large studies on colorectal cancer [4, 5]. The bottom line is that geneticists lost the interest in family-based studies once these appeared not to lead to new discoveries. However, the occurrence of rare cancers in multiple family members is hard to explain by causes other than Mendelian inheritance. Examples that family-based approaches work in the NGS era are the detection of TERT promoter and POT1 mutations in melanoma pedigrees, of POLE, POLD1 and FAN1 mutations in colorectal cancer families, KDR mutations in Hodgkin lymphoma families and of HABP2 mutations in nonmedullary thyroid cancer pedigrees [6–11]. However, several authors have pointed out that the HABP2 variant is a common polymorphism [12].

In the present article, we describe a gene identification pipeline for germline mutations in cancer families with the focus on pedigree and functional annotation data, familial cancer variant prioritization pipeline (FCVPP). The advantage of the pedigree approach is that with a decent number of affected and unaffected family members the number of candidate mutations can be drastically reduced before feeding the data on in the distal pipeline for evaluation of the likelihood for the variant of being deleterious through functional annotation.

## Methods

### Whole-exome/genome sequencing and mapping

Whole-exome/genome sequencing for the cases and controls from different families considered into the current study was performed after DNA isolation from blood samples using Illumina-based small read sequencing. Mapping of reads to reference human genome (assembly version Hs37d5) was performed using BWA [13] and duplicates were removed using Picard (http://broadinstitute.github.io/picard/).

### Variant calling and annotation

Variants were detected after mapping by using SAMtools for single nucleotide variants (SNVs) [14] and Platypus for indels [15]. Variants were annotated using ANNOVAR [16], 1000 Genomes [17], dbSNP [18] and ExAC [19].

### Variant filtering

Variants were filtered with the quality score greater than 20 and greater than 5x coverage. SNVs that passed the strand bias filter (a minimum one read support from both forward and reverse strand) and indels that passed all the Platypus internal filters were evaluated further. Minor allele frequencies (MAFs) were examined with respect to the 1000 Genomes Phase 3 and non-TCGA ExAC data [19]. We used 0.1 % MAF cut-off for rare variants deduced from these two datasets. A pairwise comparison of shared rare variants among cohort was performed to check for sample swaps and family relatedness.

### Segregation in pedigrees

The variants were filtered separately in each family based on the pedigree data by considering cancer patients as cases and unaffected persons as controls, and by applying to each individual a probability consideration for being a Mendelian case or a true control. However, as a rule of thumb was that a maximum number of cases and a minimum number of controls in each family must carry the variant.

### Variant ranking

We first ranked the variants using the combined annotation dependent depletion (CADD) tool v1.3 [20]. Any variant with the scaled PHRED CADD score of >10 belongs to top 10 % of probable functional variants and is considered deleterious, while the top 1 % and top 0.1 % variants will have CADD scores of >20 and >30, respectively [20]. All variants with CADD score >10 were taken into further consideration.

### Conservational screening of variants

To evaluate evolutionary conservation of a particular variant, two tools were used, the Genomic Evolutionary Rate Profiling (GERP [21]) and the PhastCons [22]. The GERP score of >2.0 and the PhastCons score of >0.3 indicate a good level of conservation of the variants and these scores were considered in the screening of variants.

### Evaluation of deleterious nature of the coding variants

All missense variants were assessed for deleteriousness using four tools, namely MutationTaster [23], PolyPhen V2 [24], Provean [25] and SIFT [26]. These data were gathered using dbNSFP [27]. Variants predicted to be deleterious by at least three of these tools were analysed further. Additionally, three different intolerance scores were employed to evaluate intolerance of the genes against functional mutations. These three intolerance scores were derived from our in-house datasets and from the ESP [28] and the ExAC [19].

### Prediction of deleterious nature of the non-coding variants

The regulatory nature and the possible functional effects of non-coding variants were evaluated using CADD v1.3 [20], HaploReg V4 [29] and Regulome DB [30], which are based mainly on the ENCODE data [31]. For non-coding regions variants from 127 cells from the NIH Roadmap Epigenomics Mapping Consortium were accessed via CADD v1.3 [20].

### miRNA target prediction for the 3’ UTR variants

The miRanda tool was used for finding putative miRNA targets among the 3’ UTR variants; mirSVR score lower than −0.1 is indicative of a “good” miRNA target [32].

### Visualization of variants of interests

Variant positions were visualized in the human genome using the Locuszoom [33], SNiPA [34] and the UCSC genome browser [35].

## Results

The a priori success of a pedigree-based study depends on the number and type of the available samples from family members and the population prevalence of the cancer under study, as for common cancers the likelihood of phenocopies (individuals not sharing the causative mutation) is higher than for rare cancers. For assessment of the likelihood of Mendelian inheritance, it is necessary to critically consider the pedigree data, particularly regarding diagnostic accuracy. Cancers in each of several generations would be suggestive of Mendelian inheritance. Samples from distant relatives diagnosed with the same phenotype who share a small proportion of their variants are more powerful than those from close relatives diagnosed with the same phenotype who share much of their variants. Healthy non-carriers as controls are very useful, particularly if they have passed the common diagnostic age in the family. In practice, however, true non-carriers are difficult to obtain because genetic counseling firstly considers siblings and offspring of index cases who are young for the cancer. Thus, the pedigree data can often be divided to likely and probable Mendelian cases and likely and probable non-carriers, which needs to be considered in the analysis. It is even possible to apply formal linkage analysis programs based on the NGS data.

Blood samples from members of families are often collected over a long period and many persons from various medical centers may be involved. For a start of a sequencing project, it is necessary to have a detailed pedigree with birth years, diagnostic years and, for healthy individuals, the last medical contacts. The complexities of data collection imply that errors may occur. It is possible to verify the pedigree data by analyzing genetic sharing of the individuals and thus deduce their relatedness. As errors in pedigree assignments may be fatal to the study, a simple check on relatedness is more than worth the effort before the sequence data enters the pipeline.

**Fig. 1:**
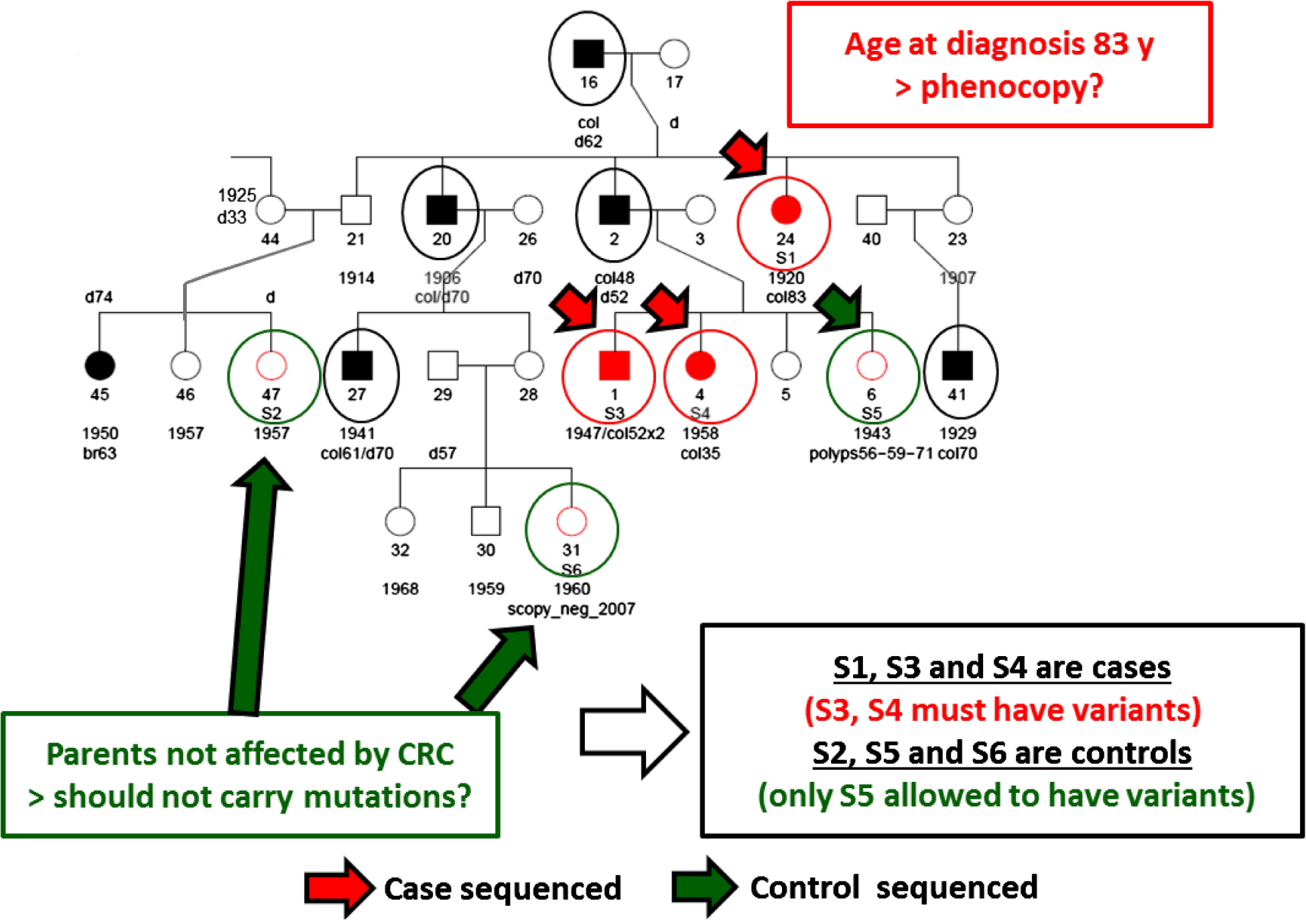
A pedigree of a high-risk colorectal cancer (CRC) family for which the individuals with arrows were exome sequenced. The consideration of cases as gene carriers and healthy individuals as non-carriers is shown in the text boxes and discussed in the text

We show a pedigree of a colorectal cancer (CRC) family that we have exome sequenced (Fig. 1). Samples were available from 3 cases and 3 healthy individuals. The cases were siblings and their paternal aunt. The CRC cases in the family (marked ‘col’) numbered 8 and there were cases in 3 generations so it qualified for a Mendelian family. However, the aunt (S1) was diagnosed at age 83 years and in the analysis we considered the possibility that she was a phenocopy. Among the healthy individuals, S5 had had polyps on 3 occasions and as she had 3 first-degree relatives with CRC we considered possible that she was a carrier. The parents and siblings of S2 and S6 had no CRC, we thus considered that they were non-carriers.

Before describing the pipeline, we show in Table 1 what happened to the likely exonic and UTR variants in the above family when the pipeline was applied. After filtering for common variants (MAF <0.1 %) and sequencing and mapping artifacts, 2920 missense variants, 934 variants at 5’UTR and 5464 variants at 3’UTR were identified. The pedigree data reduced the number of missense variants to 257, and the further functional annotation steps in the pipeline reduced the number to 10. The reduction was also marked in UTRs; the numbers in parenthesis in the last lines for UTRs show the variant numbers if intolerance scores are not considered.

**Table 1:**
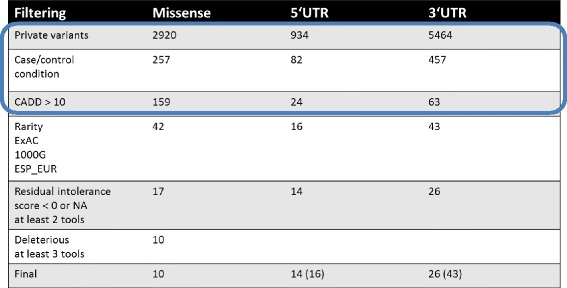
Reduction of exome sequence variants in the course of application of various conditions in the germline sequencing pipeline. The numbers in parenthesis in the last line for UTRs show the variant numbers if intolerance scores are not considered

**Fig. 2:**
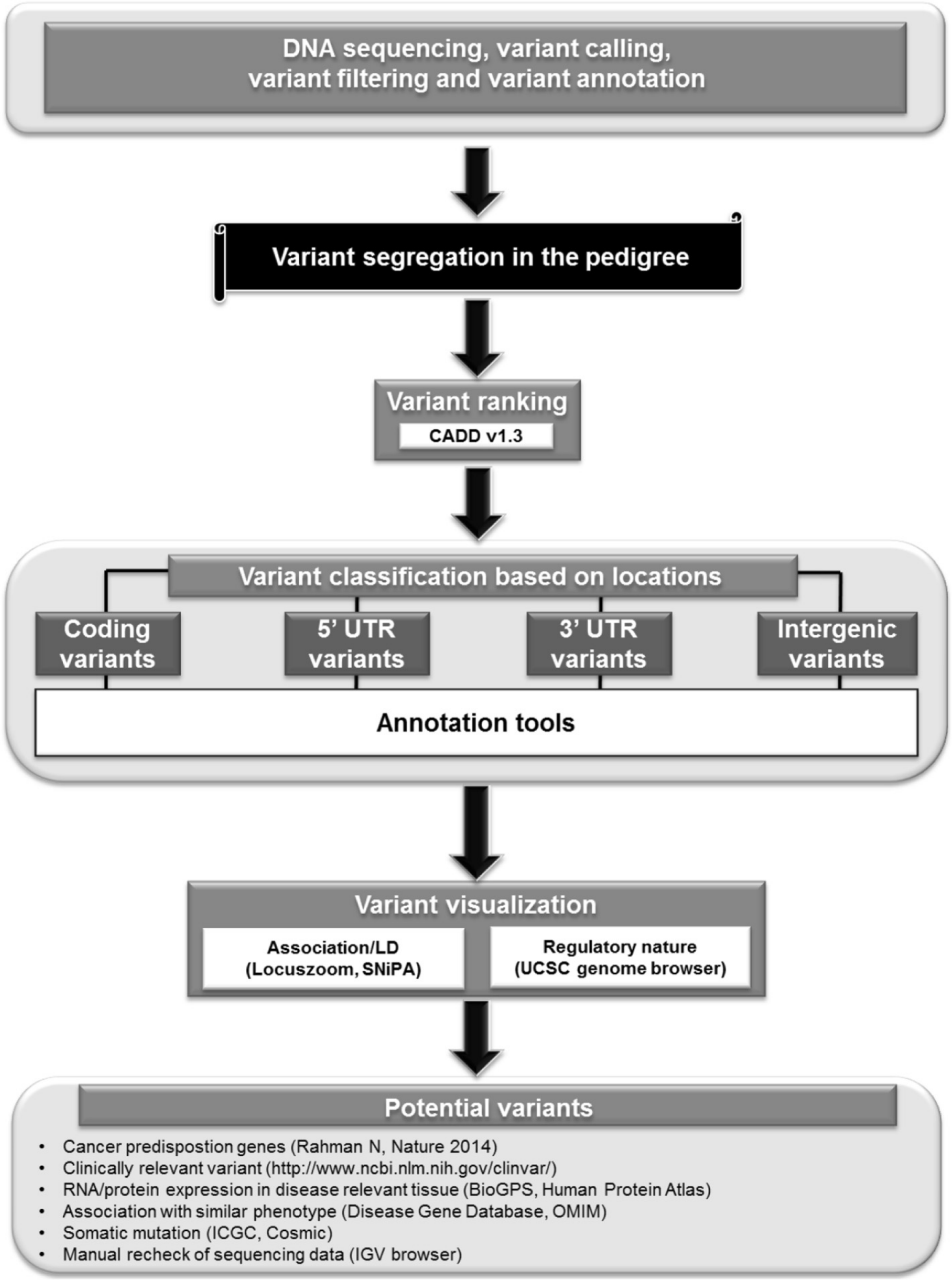
Diagram of the germline sequencing pipeline showing the technical processes on top, followed by segregation in the family, variant ranking and annotation tools based on the genomic locations of the variants

The developed pipeline is shown in Fig. 2. The initial part does not differ whether whole-exome or whole-genome sequencing is done. The proximal pipeline starts with technical parts, variant calling for single nucleotide variants (SNVs), and indels followed by variant annotation and filtering modules. Although we here focus on SNVs and indels, our pipeline is also capable of handling copy number variants (CNVs). Variant frequency data are becoming quite covering for exon sequences (1000 Genomes, EVS 6500 and ExAC datasets) while even for UTRs and more so for intronic and intergenic sequences the data on variant frequencies are still sparse, and we rely on 1000 Genomes and in-house controls.

The main focus of the pipeline is on the use of pedigree information and the functional annotation of the potential causing variants in each pedigree. In the second step the sequence data are adjusted based on the pedigree information. The basic tenet is that all or most cases should share the harmful variant which should be lacking from controls. Depending on the number and type of family members that were sampled the number of variants that pass to the next step may decrease by a factor of 10 or more (see Table 1). The remaining variants pass to the first functional annotation step, the CADD analysis, which gives a deleteriousness score based on a number of commonly used in silico tools. CADD may be very discriminatory for coding variants but may be less powerful for UTRs and intergenic variants. This is due to limited information available for non-coding regions of the human genome.

**Fig. 3:**
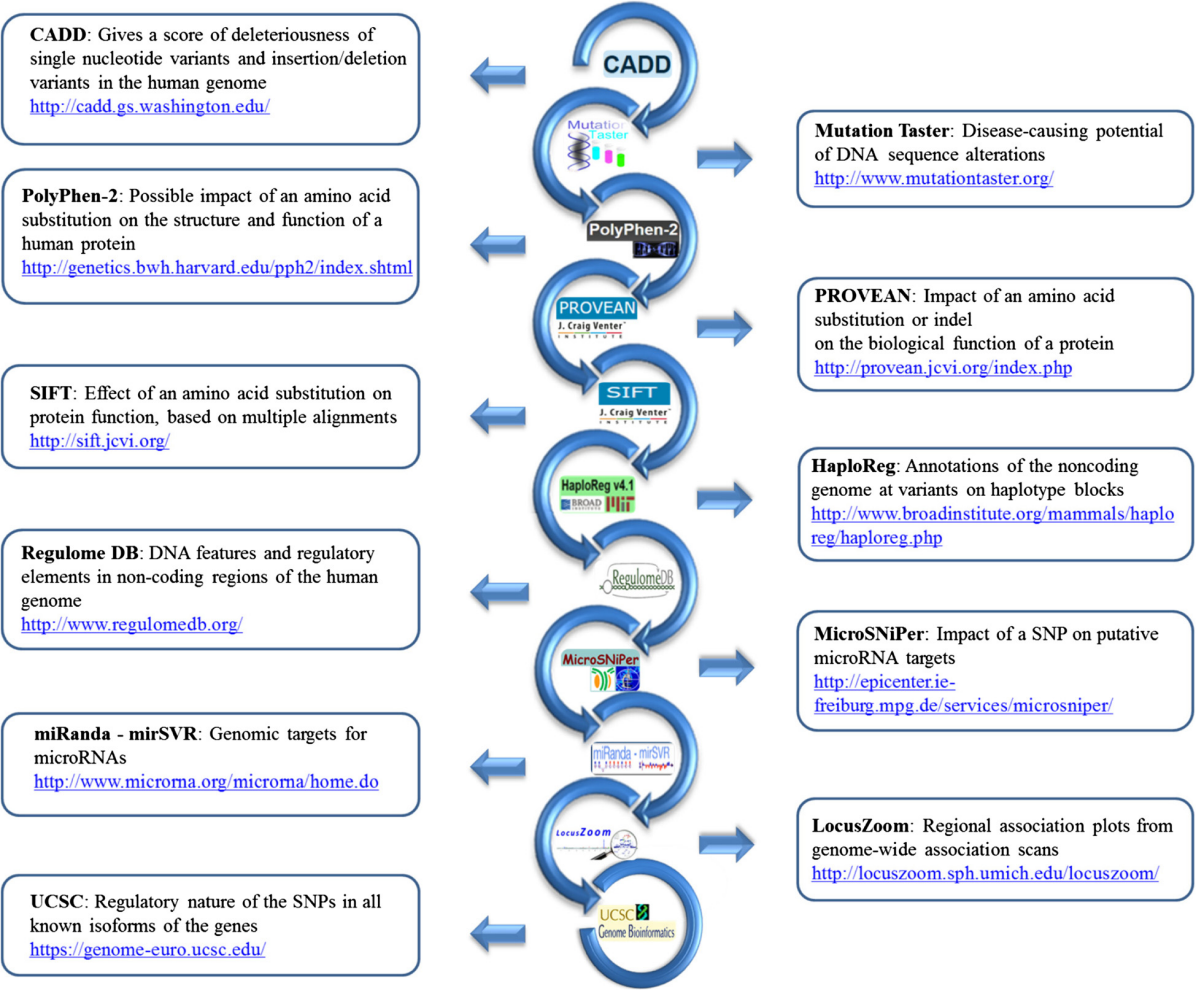
Data content of the commonly used annotation tools and their access links

After CADD the pipeline branches into 4 distal parts: coding variants, 5’ UTR, 3’ UTR and intergenic regions. Each of these regions requires a different kind of analysis and a thorough investigation of individual tools is taking place. For coding variants several tools are able to predict the deleterious nature of the variants. The analysis of UTR and intergenic variants resorts to rapidly expanding datasets such as Haploreg [29], Regulome DB [30], miRanda [32] and MicroSNiper [36]. The genomic environment of the variants can be visualized by tools such as Locuszoom, SNiPA or annotations available in the UCSC browser [35]. A short description of the available databases and their addresses are given in Fig. 3.

At the distal end of the pipeline a successful gene finder has a candidate or a few of them. We can search for confirmation in databases shown under ‘Potential variants’ in Fig. 2. Comparison with the lists of cancer predisposing genes, clinically relevant variants or association with a similar phenotype may help to identify the harmful variant. Information on somatic mutations may be useful for germline data because there is an overlap in genes manifesting both somatic and germline variants.

## Discussion

The accumulating data on germline variation in various cancers are able to explain between 15 and 50 % of the known familial risks [37]. The genetic data derive essentially from 3 different sources: family studies which have identified the majority of the known high-risk genes (over 110 genes), genome-wide association studies (GWASs) which have detected close to 400 low-penetrance genes/loci and finally the analysis of GWAS data for familial clustering, which has produced heritability estimates [37]. The proportion of the 3 sources of data contributing to various cancers differs greatly. The germline architecture of breast and ovarian cancer has a major contribution from the high-risk genes while for prostate and lung cancer the major contribution is from low-risk genes. GWAS estimates for heritability were strongest for esophageal cancer (38 %, Asian population), prostate cancer (38 %), and testicular cancer (30 %) [37].

Several tools have been developed, which integrate the pedigree information to the general sequencing pipeline (Table 2). Most tools focus on WES, although some include tools for evaluation of the non-coding variants as well [38]. Only one tool accounts for incomplete penetrance and locus heterogeneity [39]. The functionality of the tools have been tested either using simulated pedigrees or pedigrees with known mutations.

**Table 2:** Summary of tools for germline variant prioritization in pedigrees

Tools	Details	References
Familial cancer variant prioritization pipeline (FCVPP)	Gives guidelines for identification of disease causing variants based on segregation in the family pedigrees of cancer and in silico predictions for deleteriousness of all types of variants in whole-genome data. Evaluates each family individually based on phenotype and sample availability from the family members.	Current article
VAR-MD	Provides a ranked list of variants using Mendelian inheritance models, predicted pathogenicity annotation based on evolutionary sequence conservation and allele frequency data for small Mendelian-type of families with whole-exome data.	Sincan et. al. (2012) [41]
KGGSeq	Combines gene (identity-by-descent, linkage, inheritance model), variant (allele frequency, non-synonymous, disease-causing) and knowledge (protein-protein interaction, biological pathway, phenotype) level information to prioritize exome variants in disease families.	Li et. al. (2012) [42]
Annotate-it	Integrates data of coding variants, genes and samples from different sources providing filtering options for e.g. pedigree data.	Sifrim et. al. (2013) [43]
FAVR (Filtering and Annotation of Variants that are Rare)	After variant annotation, filtering for rare and likely deleterious coding variants according to in silico tools; pedigree information is used at the end step.	Pope et. al. (2013) [44]
PriVar	After variant annotation, filtering for deleterious variants based on several in silico tools, at the end different family-based criteria (e.g. linkage, inheritance model).	Zhang et. al. (2013) [45]
VariantDB	Integrates sample (e.g. family-based inheritance models) and variant (e.g. allele frequency, pathogenicity and function) annotations from diverse tools and provides gene and family/cohort based filtering possibilities.	Vandeweyer et. al. (2014) [46]
pVAAST (pedigree-Variant Annotation, Analysis and Search Tool)	A VAAST implementation for family-based data based on the composite likelihood ratio test (CLRT_v_) combines linkage analysis, allele frequency differences for cases vs. controls and phylogenetic conservation and biochemical function of the variant; takes incomplete penetrance and locus heterogeneity into account. Gives a ranking of genes/variants.	Hu et. al. (2014) [39]
FamAnn (Family Annotation)	After variant annotation of whole-genome data uses pedigree data to provide variants segregating in the family. Provides in silico predictions for deleteriousness in excel format to user for further prioritization. No recommendations for downstream prioritization strategies are provided.	Yao et. al. (2014) [38]
BiERapp	Integrates pedigree information with in silico predictions for exome variants.	Aleman et.al. (2014) [47]
FamPipe	Provides annotation of variants shared by affected family members using imputation identity-by-descent, linkage and disease model identification modules, however requires user-provided data for population allele frequencies and functional annotation of the variants for variant prioritization.	Chung et. al. (2016) [48]

Compared to the published tools, our pipeline takes into account the reality of the genetic counseling practice: incomplete family pedigrees. We consider the probability of each available family member for being a Mendelian case or a true control, separately for each family. Our pipeline also allows analysis of non-coding variants using state-of-art tools. Whether the present pipeline leads to a discovery of cancer predisposition genes does not depend on the pipeline itself but what is fed in, i.e., the pedigree data. The numbers of true Mendelian cases is as critical as is the correctness of diagnoses. A false assignment of a phenocopy as a Mendelian case or mixing of individuals or samples may have devastating consequences for the analysis. The quality of sequence data and sufficient coverage are important but not as crucial as for tumor DNA because many family members are sequenced and the sequence data should be identical between close relatives along large chromosomal segments and even whole chromosomes. We have been using the pipeline on genomic DNA but NGS does work also on paraffin embedded material, the use of which may not be as critical as it is for somatic sequencing for the above reasons.

Sooner or later comes also the ultimate question of functional effects and mechanisms. Thus, the other important point of the pipeline is the functional annotation of the potential causal variants. As an initial filtering we use here the CADD score, which combines data from different sources. For different regions of the genome, i.e. coding regions, 5’UTRs, 3’UTRs and non-coding regions, consideration of specific individual tools are important for the best possible evaluation of the deleteriousness of the variants. Finally, validation in other cancer families is warranted and population frequencies of the variants need to be determined as well as the cancer risk conveyed by the risk allele.

In clinical oncology, familial cancer has attained a prominent role because of the success in implementation of genetic testing and screening methods for known rare, hereditary cancer syndromes [40]. For patients and their family members family history may offer an explanation, however, additional knowledge about a deleterious mutation in a family may provide targeted prevention opportunities for mutation carriers and relief of anxiety for healthy family members. Our family-based WGS pipeline provides a tool to reach this goal.

## Conclusions

In summary, the present pipeline incorporates the pedigree data and various state-of-art data to annotate the variants and genes to find a causal cancer predisposition gene. Both the coverage of the human genome data and the biological understanding of its functional domains increase with a great speed implying that the pipeline as presented is continually updated to improve its performance. However, the critical bottleneck remains in the availability of informative pedigrees.
